# The complete chloroplast genome of *Saccharina latissima*

**DOI:** 10.1080/23802359.2020.1825999

**Published:** 2020-10-05

**Authors:** Xiao Fan, Weifeng Xie, Yitao Wang, Dong Xu, Xiaowen Zhang, Naihao Ye

**Affiliations:** a National Demonstration Center for Experimental Fisheries Science Education Shanghai Ocean University, Shanghai, China; b Key Laboratory of Exploration and Utilization of Aquatic Genetic Resources, Ministry of Education, Shanghai Ocean University, Shanghai, China; c International Research Center for Marine Biosciences, Ministry of Science and Technology, Shanghai Ocean university, Shanghai, China; d Yellow Sea Fisheries Research Institute, Chinese Academy of Fishery Sciences, Qingdao, China; e Function Laboratory for Marine Fisheries Science and Food Production Processes, Qingdao National Laboratory for Marine Science and Technology, Qingdao, China

**Keywords:** *Saccharina latissima*, complete chloroplast genome, Illumina sequencing

## Abstract

*Saccharina latissima* is a brown algal (class Phaeophyceae) belonging to the family Laminariaceae. We reported the *de novo* assembly and the annotation of the complete chloroplast genome of *S. latissima*. The circled cpDNA of *S. latissima* is 130,619 bp in length with a large and a small single-copy region (LSC and SSC), separated by two copies of inverted repeats (IRa and IRb). The genome contains 139 protein-coding genes (PCGs), 3 kinds of ribosomal RNAs (rRNAs), and 29 transfer RNAs (tRNAs) genes that are typical of *Saccharina* cpDNA. A phylogenetic analysis strongly supported the close phylogenetic affinity of *S. latissima* and *Saccharina japonica*. The complete cpDNA of *S. latissima* will provide valuable molecular data for further analysis of evolutionary and conservation genetic resources.

*Saccharina latissima*, a species that is distributed in Europe and North America, is of utmost ecological and increasingly economic in coastal areas (Stévant et al. 2017). As one of the domain species in oceanic forest, it plays an important role in providing shelter and nursey for numerous animals and purifying the offshore seawater(Christie et al. 2009). It belongs to the brown algae (Phaeophyceae) in the order Laminariales, an economic group that has been widely cultivated (Casado-Amezúa et al. 2019). Recently, the disappearance of the domain kelp species in European ocean forest (patterns of *S. latissima* recruitment) and degradation of germplasm in kelp culture caused by the close breeding was reported, indicating much work needs to be done on conservation genetics. Despite the mitochondrial genomes of *Saccharina* genus were recently analyzed (Wang et al. 2015), to our knowledge, the chloroplast genome has never been reported for *S. latissima*. In fact, so far only one cpDNA was uncovered in *Saccharina* genus (Wang et al. 2013). We here report the assembly of the complete cpDNA of *S. latissima* to supplement data resources for researches of evolutionary and genetic conservation.

The sample of *S. latissima* was collected from Germany. Both the specimen and DNA (NO. *Saccharina-*Ye-C14) are deposited in the herbarium of Yellow Sea Fisheries Research Institute (YSFRI), Qingdao, China. Genome data were obtained by Illumina high throughput technology (see our previous re-sequence project of *Saccharina* genome) (Ye et al. 2015). Illumina paired reads of *S. latissima* were mapped to the *S. japonica* chloroplast genome (NC_018523, 130,584 in length) using BWA, which produced the SAM file to the following step for generating the consensus using Genious (http://www.genious.com). No gap was found in the mapping result. The finished sequence was also validated by mapping the raw PE reads back to itself. BLAST (Lobo 2008), and Genious were used to perform the genome annotation, following the previous work from Wang et al. (2015).

The circled cpDNA of *S. latissima* is 1,30,619 bp in length and contains 139 protein-coding sequences (CDS), 29 transfer RNA (tRNA) genes, 3 ribosomal RNA (rRNA) genes (5S rRNA, 16S rRNA, and 23S rRNA, two copy for each). All 139 protein-coding genes (PCGs) have typical initiation codons (ATG). The overall GC content is 30.1%, which is well within the normal range of brown cpDNAs. Nucleotide frequency of the H-strand is as follows: T, 34.40%; A, 34.49%; C, 15.71%; and G, 15.39%. The chloroplast of *S. latissima* encodes 32,309 amino acids, excluding the stop codons. All the 29 typical tRNAs, ranging from 70 bp to 315 bp, possess a complete clover-leaf secondary structure. The rRNAs of the two 5S rRNAs are 109 bp and 99 bp respectively, the two 16S rRNA are 2944 bp and 2945 bp respectively, and the two copy of 23S rRNA genes are both 1479 bp.

The phylogenetic analyses of the 40 shared proteins were conducted using the maximum likehood method (Figure 1). The monophyly of three brown seaweeds (*Saccharina japonica*, *S. latissimi* and *Ectocarpus siliculosus*) and those of stramenopiles were clustered with a strong bootstrap value. The MEGA (NJ) analyses yielded the same tree topology. The plastid monophyly of cryptophyta, haptophyta and rhodophyta was significantly clustered with a support value of 90, forming a red-algal clade. Stramenopiles clade and red-algal clade have a closer phylogenetic relationship than the viridiplantae clade, which was widely supported by the secondary endosymbiosis hypothesis (Fan et al. 2020).

**Figure 1.**
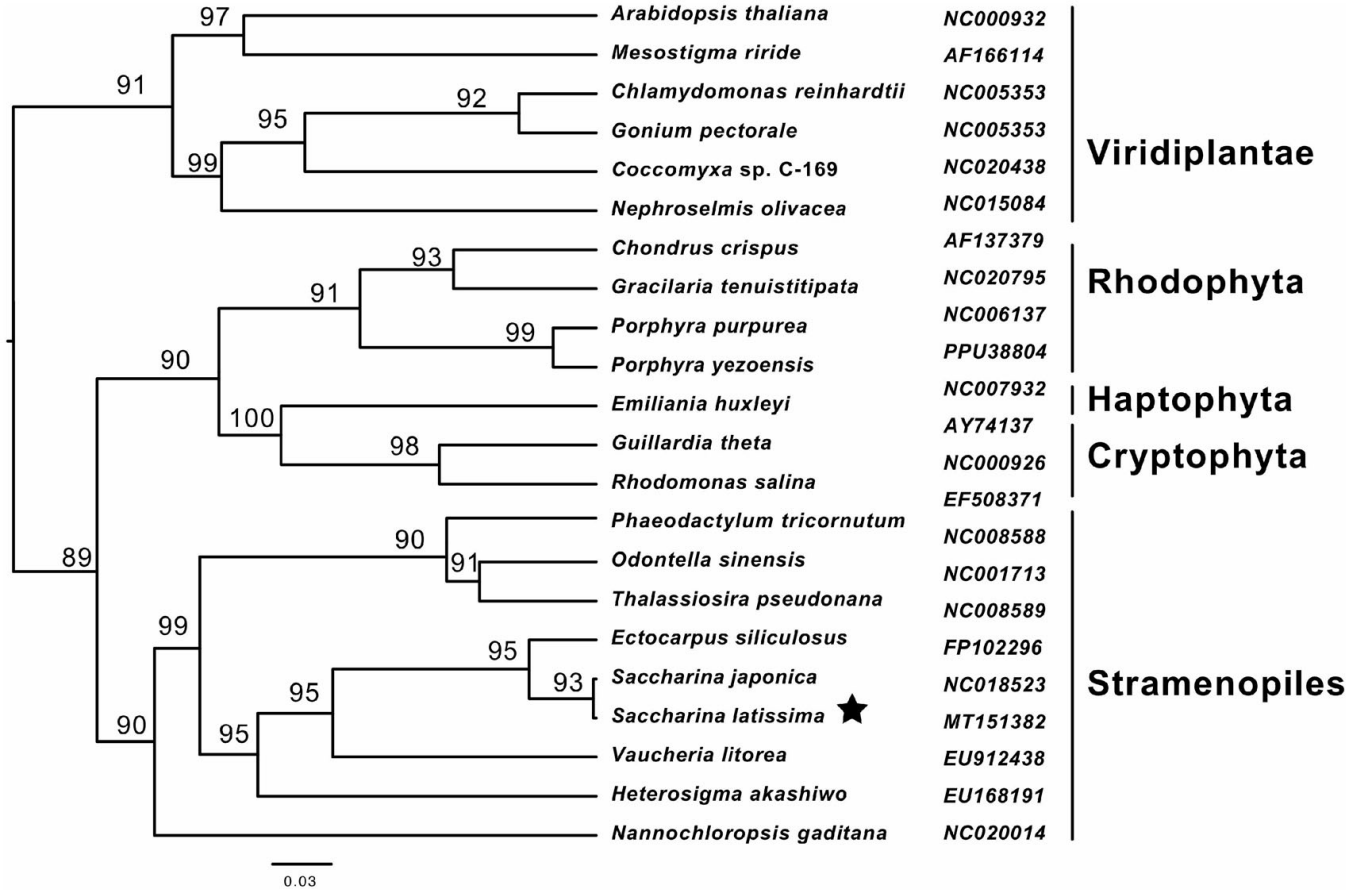
Phylogenetic tree of ML analyses based on complete chloroplast protein sequences of 22 species. Pentagrams stand for the species studied in this work.

## Data Availability

The data that support the findings of this study are openly available in NCBI GenBank with accession MT151382 or China National GeneBank DataBase (CNGBdb) https://db.cngb.org/search/sequence/N_000000748.1/.
